# Histone lysine methyltransferases KMT2C and KMT2D join the all-star tumor suppressor team in gastrointestinal cancer

**DOI:** 10.1172/JCI208392

**Published:** 2026-07-15

**Authors:** Nicole M. Peña Ruiz, Martin E. Fernandez-Zapico

**Affiliations:** 1 Division of Oncology Research and; 2 Molecular Pharmacology and Experimental Therapeutics Program, Mayo Clinic Graduate School of Biomedical Sciences, Mayo Clinic, Rochester, Minnesota, USA.

## Abstract

Members of the type 2 histone lysine methyltransferase family (KMT2s) are key drivers of enhancer activation and are the most mutated group of epigenetic regulators in different cancer types. Within this family, KMT2C and KMT2D have the highest mutational incidence across various cancers. To evaluate their role in gastric cancer, Wang et al. developed a *Pten* deficiency–driven genetically engineered mouse model with inducible loss of *Kmt2c* and *Kmt2d* in gastric epithelial cells. Through extensive in vitro, in vivo, and in silico analyses, the authors revealed that the concomitant loss of *Kmt2c* and *Kmt2d* promotes gastric carcinogenesis while enhancing antigen presentation and sensitivity to immunotherapy and targeted approaches like mTOR inhibition, highlighting the tumor-suppressive roles of KMT2C/D in gastric cancer and uncovering a vulnerability for this dismal condition.

## KMT2 family mutations may support cancer-promoting reprogramming

Type 2 histone lysine methyltransferases (KMT2s) belong to the most well-characterized family of writers of methylation of histone 3 lysine 4 (H3K4me) (1, 2). KMT2s are part of the complex proteins associated with set1–like (COMPASS-like) complex, an evolutionarily conserved 6-member group of proteins that promotes genome accessibility and transcriptional activation, in part by increasing H3K4me at genomic regulatory elements (3). Further, it has been reported that KMT2s are responsible for monomethylation of H3K4–enriched (H3K4me1-enriched) lineage-specific enhancers, which are highly dynamic distal *cis*-regulatory elements that physically loop in 3D to interact with promoter regions and activate target gene expression. The activity of these enhancers is key to defining cell type–specific transcriptional programs that are essential for maintaining cellular phenotypes (4, 5).

KMT2s, historically termed mixed lineage leukemia (MLL) proteins, were originally identified through translocations of *KMT2A* (then known as *MLL1*) at chromosome 11q23, causing oncogenic fusions that result in pediatric and adult MLL (6–8). For the past decade, the majority of genetic studies have emphasized *KMT2A*-associated somatic rearrangements in leukemia. However, a number of recent studies have identified missense, nonsense, and frameshift mutations occurring at the SET domain (the catalytic domain responsible for histone methylation) or the N-terminal domain of KMT2s in nonhematological cancers (9, 10). However, *KMT2A* is not the most abundantly mutated member of the methyltransferase family; *KMT2C* and *KMT2D* are the members with the highest mutational incidence of the COMPASS family across a broad range of gastrointestinal (GI) cancer types (1, 2, 11–15). KMT2C and KMT2D mainly mediate H3K4me1 at enhancer regions (4). Thus, as writers of the chromatin mark required for enhancer activation, KMT2C and KMT2D play a central role in multiple cellular functions (16). Aberrant KMT2C and KMT2D function could lead to epigenetic reprogramming to promote development of human cancers (17). In this issue, Wang et al. (18) further examined the *KMT2C* and *KMT2D* mutational landscape in gastric and stomach adenocarcinoma (STAD). Their findings confirmed a high mutation rate for *KMT2C* and *KMT2D* that was previously reported in STAD and identified an increased frequency of concomitant mutations in these methyltransferases.

## Combined KMT2C/D loss enhances phosphatase and tensin homolog knockout–driven cancer progression

Given the structural similarity between KMT2C and KMT2D and their overlapping roles in regulation and target gene expression, it has been suggested that concurrent loss of KMT2C/D may contribute to tumorigenesis (1, 18). Wang et al. therefore sought to characterize the epigenomic role of *KMT2C/D* loss in gastric cancer. To this end, they developed a genetically engineered mouse model (GEMM) to conditionally knock out *Kmt2c*/*d* in gastric epithelial cells, utilizing a tamoxifen-induced *Tmprss2-CreER^T2^* recombinase system (19). Histopathological analysis showed that *Kmt2c/d* loss led to nuclear dysplasia, cellular crowding, abnormal expansion of cells, and loss of gastric lineage markers. Further, the authors created a second GEMM that combined loss of the phosphatase and tensin homolog (PTEN) tumor suppressor, a negative regulator of the PI3K pathway that has been shown to promote gastric carcinogenesis (14, 19–21), with *Kmt2c/d* KO. They observed that *Pten* plus *Kmt2c/d* loss induced invasive and aggressive gastric tumorigenesis in the span of approximately 3 weeks, demonstrating that *Kmt2c/d* KO cooperates with the PI3K pathway to drive tumorigenesis by promoting an oncogenic primed molecular state and serving their role as tumor suppressors. The authors concluded that *Kmt2c/d* loss alone does not have the ability to initiate cancer, a phenomenon that was also recently described in other GI cancers (e.g., pancreatic cancer), but the loss of these KMTs can accelerate tumorigenesis only in the presence of second protumoral stimulus (e.g., *KRAS^G12D^*) (22).

## KMT2C/D loss shapes potential vulnerabilities in gastric cancer

Further analysis of this phenotype using single-cell RNA-seq (scRNA-seq) revealed that *Kmt2c/d* and *Pten* depletion upregulated MHC class I (MHC-I) expression and antigen presentation and led to inadequate translation of proteins. Similar findings have been reported in STAD (23), where *KMT2C/D* mutations lead to protein truncations, highlighting how malfunctioning KMT2C/D-driven regulation can contribute to tumorigenesis in gastric cancers. Next, the authors validated the presence of B2M, a component of MHC-I, conducted CRISPR/Cas9 KO of *Kmt2c/d* in *Pten*-deficient cells, and performed an ovalbumin antigen system in their GEMMs. With these techniques, they confirmed that the concurrent loss of *Kmt2c/d* upregulated the expression of MHC-I and significantly increased T cell–mediated cytotoxicity, suggesting that *KMT2C/D* loss shapes the immune landscape in gastric cancer and implying potential sensitivity to immunotherapy. Moreover, the scRNA-seq analysis also revealed a positive enrichment of gene sets associated with protein translation following *Kmt2c/d* deletion. To explore this finding further, Wang et al. performed drug treatments with mTOR inhibitors followed by bulk RNA-seq and identified that *Kmt2c/d* loss confers sensitivity in a paradoxical manner: mTORC1 treatment suppressed the expression of mTORC1 downstream genes, as well as triggering the upregulation of ribosomal protein (RPs), suggesting a compensatory mechanism between protein synthesis and RP expression (18).

To define how methylation states are altered by *Kmt2c/d* loss, the authors performed chromatin immunoprecipitation with sequencing (ChIP-seq) and compared the *Kmt2c/d-* and *Pten*-deficient GEMM with a *Pten*-deficient GEMM. They observed that *Kmt2c/d* KO induced a decrease of H3K4me1 at enhancers and promoters, with only a minor reduction in trimethylation of H3K4 (H3K4me3), an epigenetic modification found at actively transcribed gene promoters, at these regions. A pooled analysis of 45 RP genes showed no significant difference of H3K4me3 following *Kmt2c/d* loss. The finding suggests that the upregulation of RP genes is not a direct consequence of altered histone methylation at their promoters, which reinforces the compensatory response to inadequate translation. This phenomenon is expected since it has been well characterized that other COMPASS members, such KMT2A and KMT2B, are enriched at promoters and catalyze specifically H3K4me3 (1, 2). Additionally, previous works have shown how *Kmt2c/d* loss induces changes in chromatin states and redistributes KMT2A-menin to CpG-high promoters, demonstrating the coordinated transcriptional regulation and compensatory redistribution of the COMPASS family in the epigenome (14).

## Exploring a multidrug regimen in KMT2C/D-deficient gastric cancer

Aiming to uncover potential therapeutic opportunities, Wang et al. performed cell viability assays utilizing PI3K/AKT/mTOR pathway inhibitors and identified that *Kmt2c/d* loss confers sensitivity to mTORC1 inhibition in gastric cancer, suggesting that co-incidence of *Kmt2c/d* loss promotes a therapeutic vulnerability to mTOR complex 1 (mTORC1) inhibition. To evaluate if a multidrug regimen would enhance treatment efficacy, the authors performed a combination of mTORC1 with anti–programmed cell death 1 (anti–PD-1) immune checkpoint blockade and compared this treatment with mTORC1 and anti-PD1 monotherapies in the *Pten-* and *Kmt2c/d*-deficient GEMM. The combination therapy demonstrated synergy and led to reduced tumor weight, increased CD8^+^ T cell infiltration, improved histological differentiation, and enhanced survival compared with either monotherapy, suggesting that patients with KMT2C/D-deficient STAD may benefit from this combination regimen (18).

## Conclusion

**Figure 1:**
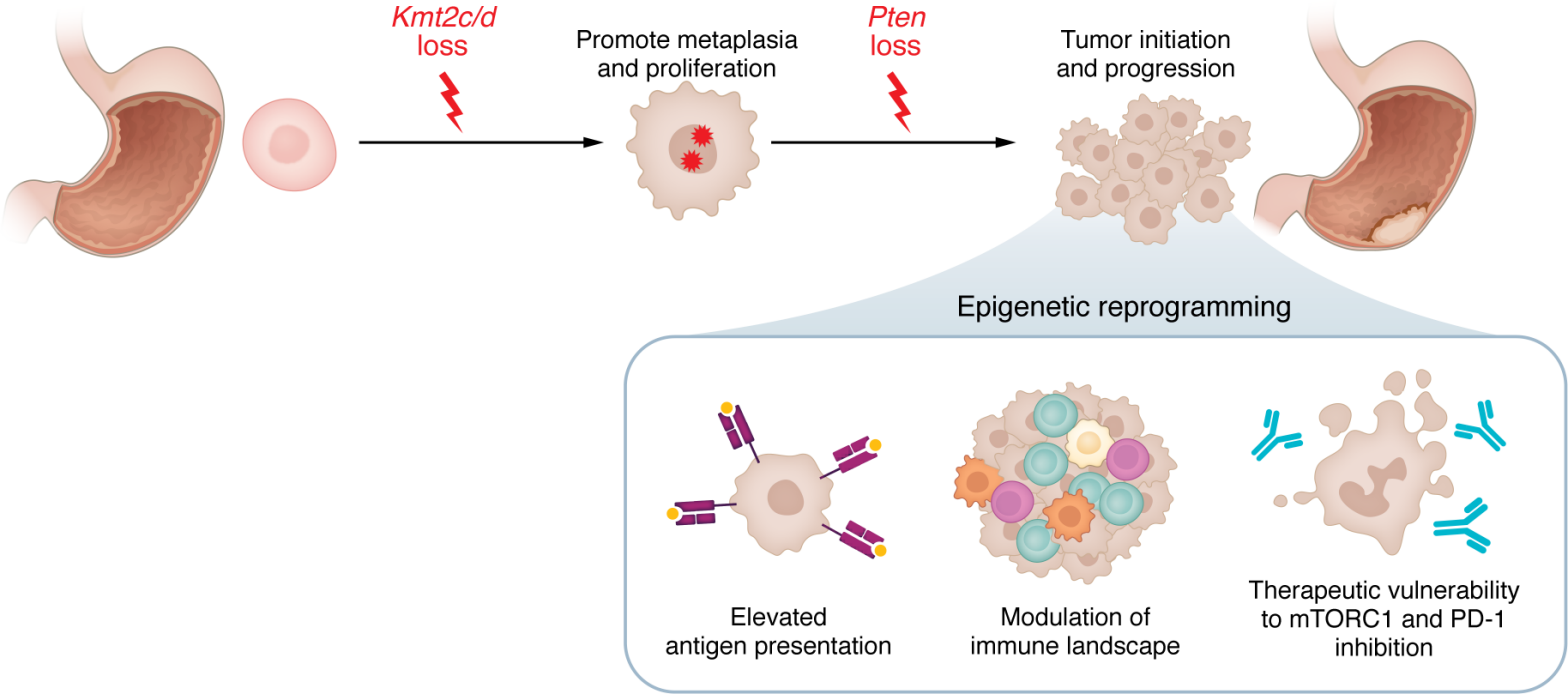
KMT2C and KMT2D are potentially novel tumor suppressors in gastric carcinogenesis. Wang et al. (19) showed that concurrent loss of *Kmt2c* and *Kmt2d* (*Kmt2c/d*) promoted metaplasia and cell proliferation as well as impaired gastric lineage differentiation. Further, combined loss of *Kmt2c/d* and *Pten* resulted in tumor initiation and progression, therefore highlighting the tumor suppressor role of these methyltransferases. Mechanistically, the KMT2C/D loss led to an altered epigenetic landscape, increasing antigen presentation, modulating the tumor immune profile, and conferring therapeutic vulnerabilities to immunotherapy (PD-1 inhibitor) combined with targeted therapy (mTORC1 inhibitor).

This work defines the role of *KMT2C/D* loss in gastric cancer as tumor suppressive for this malignancy and demonstrates the roles of KMT2C/D as modulators of the tumor immune landscape (Figure 1). Given their prevalence in human cancers and their impact on epigenetic cellular reprogramming, mutations and loss of expression in KMT2C/D may represent an opportunity to stratify patients for treatment selection, supporting the rationale for future individualized approaches for KMT2C/D-deficient gastric cancer cases. The use of targeted next-generation sequencing panels including *KMT2C/D* in profiling panels could also provide a prognostic value for patients with gastric cancer and immunotherapy response (16). Additionally, recent studies have reported similar findings in other GI cancers (24), where *KMT2D* loss is associated with PD-L1 expression and increased infiltration of immune cells, further supporting the use of immunohistochemistry-based biomarkers to predict immune checkpoint blockade efficacy. Thus, in summary, Wang et al.’s findings support utilizing concurrent loss of KMT2C/D as a predictive biomarker for patient stratification, early detection, and combinational treatment strategies for gastric cancer to guide precision oncology approaches and target the biological dependencies driven by these enhancer regulators.

## Conflict of interest

The authors have declared that no conflict of interest exists.

## Funding support

This work is the result of NIH funding, in whole or in part, and is subject to the NIH Public Access Policy. Through acceptance of this federal funding, the NIH has been given a right to make the work publicly available in PubMed Central.

- NIH funding to MEFZ (CA265050).
- NMPR by the Mayo Clinic Comprehensive Cancer Center Fellowship.

## Version 1 07/15/2026

Electronic publication
